# Association of elevated fractional exhaled nitric oxide concentration and blood eosinophil count with severe asthma exacerbations

**DOI:** 10.1186/s13601-019-0282-7

**Published:** 2019-08-21

**Authors:** David B. Price, Sinthia Bosnic-Anticevich, Ian D. Pavord, Nicolas Roche, David M. G. Halpin, Leif Bjermer, Omar S. Usmani, Guy Brusselle, Simon Wan Yau Ming, Sarang Rastogi

**Affiliations:** 1 Observational and Pragmatic Research Institute, Singapore, Singapore; 2 Academic Primary Care, Division of Applied Health Sciences, University of Aberdeen, Polwarth Building, Foresterhill, Aberdeen, AB25 2ZD UK; 3 Woolcock Institute of Medical Research, University of Sydney, Sydney, Australia; 4 Oxford NIHR Respiratory Biomedical Research Centre, Nuffield Department of Medicine, University of Oxford, Oxford, UK; 5 University Paris Descartes, Paris, France; 6 Royal Devon and Exeter Hospital, Exeter, UK; 7 Lund University, Lund, Sweden; 8 Imperial College London, London, UK; 9 Ghent University Hospital, Ghent, Belgium; 10 Global Medical Affairs, AstraZeneca, Gaithersburg, USA

**Keywords:** Asthma, Blood eosinophils, Exhaled airway markers, Nitric oxide

## Abstract

**Background:**

Blood eosinophil count (BEC) and fractional exhaled nitric oxide (FeNO) concentration are established biomarkers in asthma, associated particularly with the risk of exacerbations. We evaluated the relationship of BEC and FeNO as complementary and independent biomarkers of severe asthma exacerbations.

**Methods:**

This observational study included data from the Optimum Patient Care Research Database. Asthma patients (18–80 years) with valid continuous data for 1 year before FeNO reading, ≥ 1 inhaled corticosteroid prescription, and BEC recorded ≤ 5 years before FeNO reading were separated into cohorts. Categorisation 1 was based on the American Thoracic Society criteria for elevated FeNO concentration (high: ≥ 50 ppb; non-high: < 25 ppb) and BEC (high: ≥ 0.300 × 10^9^ cells/L; non-high: < 0.300 × 10^9^ cells/L). Categorisation 2 (FeNO concentration, high: ≥ 35 ppb; non-high: < 35 ppb) was based on prior research. Reference groups included patients with neither biomarker raised.

**Results:**

In categorisation 1, patients with either high FeNO or high BEC (n = 200) had a numerically greater exacerbation rate (unadjusted rate ratio, 1.31 [95% confidence interval: 0.97, 1.76]) compared with patients in the reference group. Combination of high FeNO and high BEC (n = 27) resulted in a significantly greater exacerbation rate (3.67 [1.49, 9.04]). Similarly, for categorisation 2, when both biomarkers were raised (n = 53), a significantly greater exacerbation rate was observed (1.72 [1.00, 2.93]).

**Conclusion:**

The combination of high FeNO and high BEC was associated with significantly increased severe exacerbation rates in the year preceding FeNO reading, suggesting that combining FeNO and BEC measurements in primary care may identify asthma patients at risk of exacerbations.

**Electronic supplementary material:**

The online version of this article (10.1186/s13601-019-0282-7) contains supplementary material, which is available to authorized users.

## Background

Asthma, a chronic inflammatory disorder of the airways affecting more than 315 million people worldwide, is associated with considerable morbidity, mortality, and loss of productivity [1–3]. Recognised as a complex, heterogeneous disease, asthma is associated with several phenotypes [4]. Approximately 50% of all asthma patients demonstrate evidence of eosinophilic airway inflammation [5, 6], which is associated with an increased risk of exacerbations [7, 8]. Severe asthma exacerbations involve systemic corticosteroid use, emergency room visits, and/or hospitalisations [9, 10]. Therefore, an important goal in the treatment and management of asthma is preventing exacerbations by identifying patients most at risk.

Blood eosinophil counts and fractional exhaled nitric oxide (FeNO) concentrations are established biomarkers in asthma. A high blood eosinophil count, used as a marker for eosinophilic airway inflammation, correlates well with poor asthma control, an increased risk of severe exacerbations, and re-hospitalisations [11–14]. Conversely, a significant reduction in severe exacerbations has been observed for severe asthma patients with elevated blood eosinophils treated with biologics targeting type 2 cytokines involved in eosinophilic inflammation [15–18]. A FeNO concentration greater than 50 parts per billion (ppb) is a marker for eosinophilic airway inflammation and predicts the likelihood of corticosteroid responsiveness [19, 20]. Moreover, elevated FeNO is considered a risk factor for exacerbations in adult asthma patients prescribed inhaled corticosteroids (ICS) [21, 22]. Therefore, measurement of FeNO may provide additional predictive value to blood eosinophil counts for severe exacerbations in asthma patients.

Although both blood eosinophil count and FeNO concentration are associated with eosinophilic airway inflammation, they demonstrate only a modest correlation, reflecting different parts of the T2-driven inflammation [23–26]. Notably, these biomarkers vary in their responsiveness to and ability to predict response to biologic therapy for asthma [16, 27, 28].

Anti-interleukin-5 treatment with mepolizumab lowered blood eosinophil counts without affecting FeNO concentrations [28], while blocking interleukin-13 with lebrikizumab reduced FeNO concentrations without affecting blood eosinophil counts [27]. Thus, FeNO may also reflect aspects of T2-driven inflammation not directly related to eosinophils. While strong evidence suggests that ICS treatment has a substantial effect on FeNO readings, sparse evidence supports the dose–response effect of ICS on blood eosinophil counts [24, 25]. Presence of raised FeNO concentrations and raised blood eosinophil counts, despite adherence to treatment, may identify patients with poor sensitivity to ICS who require a more targeted, personalised approach to therapy. Therefore, identification of a phenotype that demonstrates raised blood eosinophil counts and/or FeNO concentrations, despite ICS therapy, could be valuable. The aim of this study was to determine whether FeNO concentration added value to blood eosinophil counts for identification of patients at risk of asthma exacerbations. We, therefore, retrospectively analysed data from a large validated national database of patients in the United Kingdom (UK) to evaluate whether a high blood eosinophil count combined with high FeNO concentration was associated with an increased risk of severe asthma exacerbations.

## Methods

### Data source and study design

This cross-sectional study was conducted using patient data from the Optimum Patient Care Research Database (OPCRD). The OPCRD is a primary care database containing high-quality anonymised data obtained from longitudinal medical records and patient-completed questionnaires in the UK health care system [29]. Patient data were assessed for 1 year preceding the index date (baseline year). The study was registered under the established study database, namely, the European Network of Centres for Pharmacoepidemiology and Pharmacovigilance (registration number: EUPAS16891). Ethical approvals were obtained from the Anonymised Data Ethics and Protocol Transparency committee [30].

**Fig. 1:**
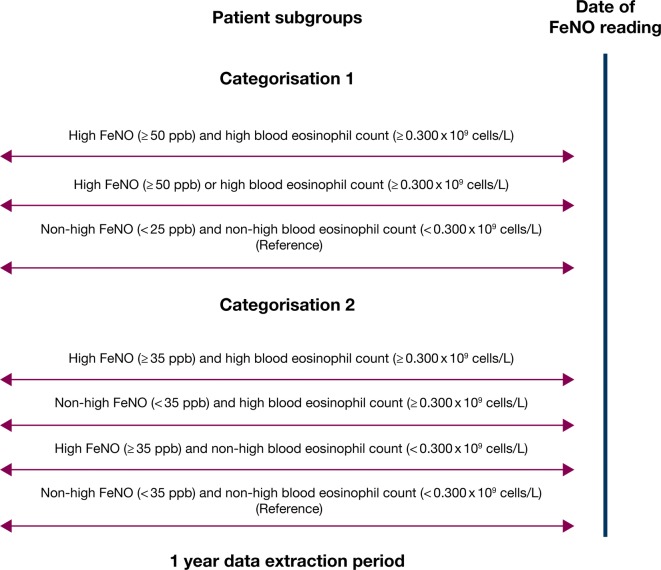
Study design. The study period consisted of the year prior to the latest documented FeNO reading. *FeNO* fractional exhaled nitric oxide

Patients were classified based on their FeNO reading on the index date and the closest blood eosinophil count reading (Fig. 1). Two sets of thresholds were used for FeNO: (1) based on the American Thoracic Society (ATS) [19] criteria defining high FeNO (≥ 50 ppb), medium FeNO (25 to < 50 ppb), and low FeNO (< 25 ppb) concentrations (categorisation 1); and (2) based on previous research [31, 32] suggesting poor asthma control with FeNO concentrations ≥ 35 ppb, high FeNO was defined as ≥ 35 ppb and non-high FeNO, < 35 ppb (categorisation 2). In both categorisation schemes, the cutoff to define a high blood eosinophil count was set at ≥ 0.300 × 10^9^ cells/L. Categorisation 1 included three cohorts: high FeNO (≥ 50 ppb) and high blood eosinophil count (≥ 0.300 × 10^9^ cells/L), high FeNO alone (≥ 50 ppb) or high blood eosinophil count alone (≥ 0.300 × 10^9^ cells/L), and non-high FeNO (< 25 ppb) and non-high blood eosinophil count (< 0.300 × 10^9^ cells/L) (reference group). Categorisation 2 included four cohorts: high FeNO (≥ 35 ppb) and high blood eosinophil count (≥ 0.300 × 10^9^ cells/L), high FeNO (≥ 35 ppb) and non-high blood eosinophil count (< 0.300 × 10^9^ cells/L), non–high FeNO (< 35 ppb) and high blood eosinophil count (≥ 0.300 × 10^9^ cells/L), and non-high FeNO (< 35 ppb) and non-high blood eosinophil count (< 0.300 × 10^9^ cells/L) (reference group).

The study period, during which both patient characteristics and outcomes were observed, consisted of the year prior to the latest documented FeNO reading.

### Patients

The study population consisted of patients who met the following criteria: age 18–80 years inclusive; a diagnostic Read code for asthma qualifying for inclusion in the asthma patient registry, which general practices in the UK maintain for the Quality Outcomes Framework [33]; active asthma with ≥ 1 prescription for asthma medication, including ICS in the year prior to the index date; ≥ 1 valid blood eosinophil count recorded without a recent exacerbation (within 2 weeks) at most ≤ 5 years before FeNO reading; and valid continuous data for 1 year prior to the latest FeNO reading.

Patients were excluded from the study if they had a diagnosis Read code for chronic obstructive pulmonary disease or any chronic respiratory disease other than asthma; received a long-acting muscarinic antagonist or were prescribed maintenance oral corticosteroids (OCS); and had a forced expiratory volume in 1 s/forced vital capacity < 0.7.

### Outcomes

The primary outcome was the annual rate of severe asthma exacerbations, defined as the number of severe exacerbations in the study period per patient. A severe exacerbation was defined in line with the European Respiratory Society/ATS Position Statement [9] as an acute prescription of OCS, or an unplanned lower respiratory tract-related hospitalisation, or an accident and emergency attendance associated with a lower respiratory Read code or primary care respiratory consultation within 14 days.

Secondary outcomes included a description of demographics, lung function, comorbidities, respiratory medication use and ICS adherence for each of the patient groups characterised by biomarker concentrations. ICS adherence was defined using Medication Possession Ratio, calculated by dividing the total of 1 day’s supply by the total number of days evaluated (365 days in the study year), and expressed in percentage.

### Statistical analyses

All statistical analyses were conducted using Stata SE version 14.2 and R version 3.0.2.

The sample size was calculated by accounting for multiple testing with a Bonferroni correction. With four comparisons and an alpha significance level of 0.0125, 800 patients were initially deemed necessary to demonstrate at least a 20% difference between groups, with a 90% power. However, this was later revised to detect a difference in a single outcome only, namely, a 20% difference in exacerbation rate between two groups of interest.

Comparisons were initially unmatched for the purpose of exploring the main differences between patient groups and providing the steering committee with data in order to make a decision on which patient groups to compare. In addition, multivariate regression models were fitted to account for potential confounding of patient characteristics that may have varied between patient groups. Standardised mean difference was calculated to measure effect size. Characterisation and subsequent matched analyses of study outcomes were performed based on categorisations 1 and 2. Descriptive statistics of all characteristics were computed for each group of patients within the cohorts. Continuous variables were summarised using the number and percentage of non-missing observations, mean and standard deviation (SD) for normally distributed variables, and median and interquartile range (difference between the 25th and 75th percentiles) for non-normally distributed variables. Pearson’s Chi square test was used to compare percentages between different groups, with a Fisher’s test used in cases of small numbers of observations per group. Student’s *t* test was used to compare a continuous variable between two groups, with a non-parametric Mann–Whitney test used for small numbers of observations per group. Summary statistics were presented as counts and percentages. For missing data, percentages for categorical variables were provided as a percentage of the non-missing observations. A statistically significant result was defined as a p ≤ 0.05.

The primary analysis for categorisation 1 compared the number of severe exacerbations for matched patients with a high FeNO and high blood eosinophil count with that of patients with a non-high FeNO and non-high blood eosinophil count (reference group). The rate of severe exacerbations was also compared between matched patients with a high FeNO or a high blood eosinophil count vs. the reference group. The analysis for categorisation 2 compared patients with a high FeNO and high blood eosinophil count vs. the reference group, a high blood eosinophil count alone vs. the reference group, and a high FeNO alone vs. the reference group.

Conditional Poisson regression analysis was performed to estimate the rate ratio (RR) between the groups of interest, with a 95% confidence interval (CI). Unadjusted RRs were calculated based on previous knowledge of multivariable prediction models [34, 35].

## Results

**Fig. 2:**
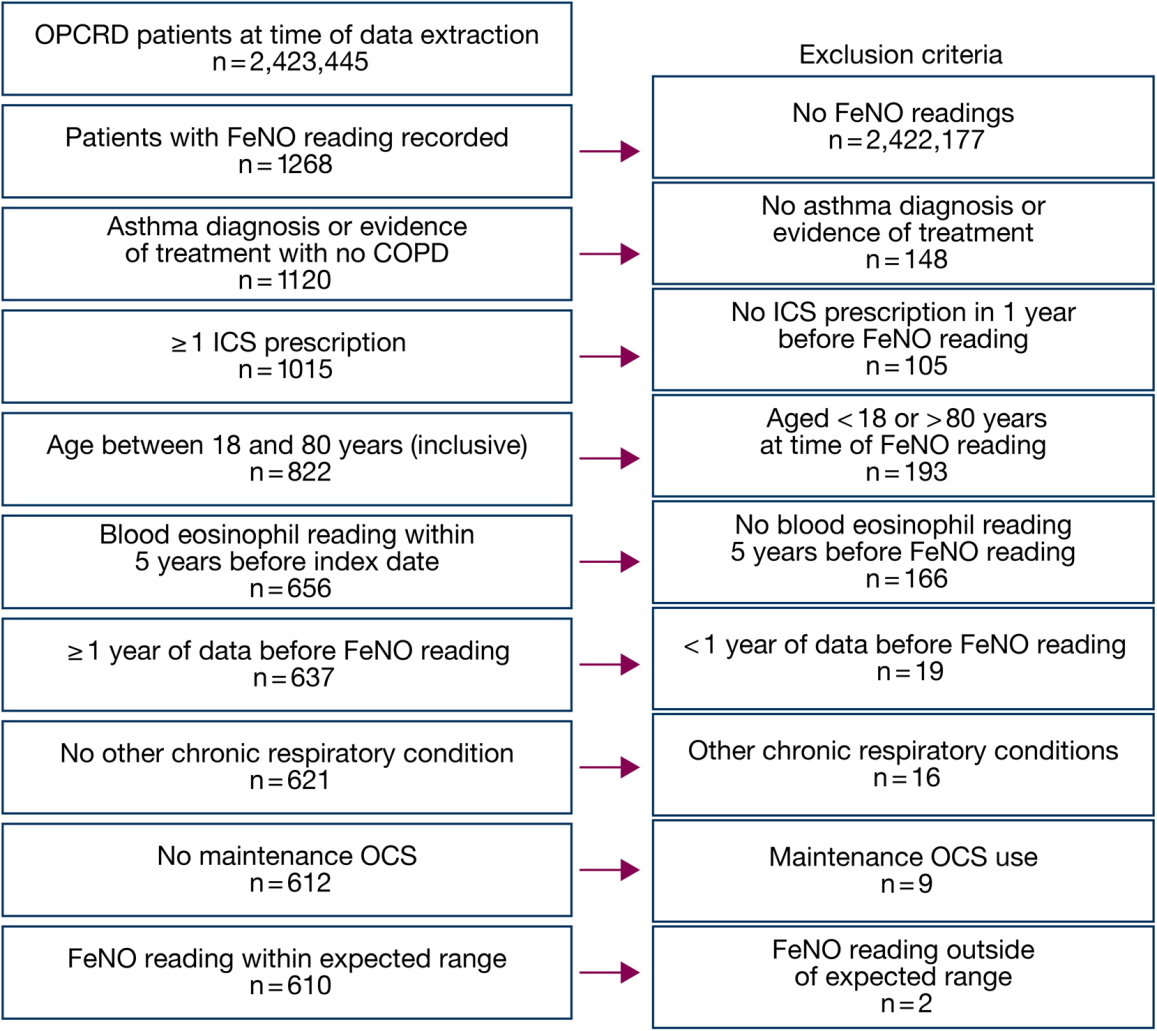
Patient selection from OPCRD. *COPD* chronic obstructive pulmonary disease, *FeNO* fractional exhaled nitric oxide, *ICS* inhaled corticosteroid, *OCS* oral corticosteroid, *OPCRD* Optimum Patient Care Research Database

At the time of the study, the OPCRD contained more than 2.4 million available patient records from more than 560 practices across the UK (Fig. 2). According to the records, 1268 patients had a recorded FeNO reading and of these, 610 patients met all other eligibility criteria and were included in the study population. Unmatched comparisons were made to assist with determining the eventual matching criteria. An additional file shows that differences were observed in sex, smoking status, body mass index (BMI), and prescription of OCS in the study year (Additional file 1: Tables S1, S2). Patients were matched 1:1 on age (within 10 years), sex, and smoking status. Further criteria to match were not included to preserve numbers in the cohort of interest.

Patients were subsequently categorised based on FeNO concentration and blood eosinophil count, such that patients from each subgroup with at least one elevated variable of interest (FeNO and/or blood eosinophil count) were matched 1:1 with the reference group (non-high FeNO and non-high blood eosinophil count).

### Demographics and baseline clinical characteristics

#### Categorisation 1

A total of 27 patients in the high FeNO and high blood eosinophil cohort matched with the reference group (Table 1). Overall, 63% of patients were female, with most patients aged 35–65 years. In addition, 51.9% of patients were non-smokers. In the second cohort, 200 patients with high FeNO or high blood eosinophil count matched with the reference group (Table 2). Overall, 58% of patients were female, with most patients aged 35–65 years. A total of 36.5% of patients were non-smokers.

**Table 1:** Categorisation 1: non-high FeNO and non-high blood eosinophils vs. high FeNO and high blood eosinophils

Characteristics	Non-high FeNO and non-high blood eosinophils (n = 27)	High FeNO and high blood eosinophils (n = 27)	p-value
Sex			
n (% non-missing)	27 (100.0)	27 (100.0)	1.0000
Male	10 (37.0)	10 (37.0)	
Age			
n (% non-missing)	27 (100.0)	27 (100.0)	0.9379
Mean (SD)	43.5 (18.8)	43.3 (19.0)	
Median (IQR)	41.0 (37.0)	41.0 (37.0)	
Age group			
n (% non-missing)	27 (100.0)	27 (100.0)	1.0000
Under 35	12 (44.4)	12 (44.4)	
35–65	9 (33.3)	9 (33.3)	
66–80	6 (22.2)	6 (22.2)	
Smoking status			
n (% non-missing)	27 (100.0)	27 (100.0)	0.7645
Non-smoker	13 (48.1)	14 (51.9)	
Ex-smoker	1 (3.7)	2 (7.4)	
Current smoker	13 (48.1)	11 (40.7)	
BMI			
n (% non-missing)	23 (85.2)	26 (96.3)	0.4346
Mean (SD)	27.7 (7.2)	26.9 (6.4)	
Median (IQR)	26.8 (5.5)	25.5 (8.4)	
Active eczema diagnosis^a^			
n (% non-missing)	27 (100.0)	27 (100.0)	0.6387
Yes	3 (11.1)	2 (7.4)	
Active rhinitis diagnosis^a^			
n (% non-missing)	27 (100.0)	27 (100.0)	0.2482
Yes	7 (25.9)	11 (40.7)	
Eczema diagnosis			
n (% non-missing)	27 (100.0)	27 (100.0)	0.7801
Yes	10 (37.0)	11 (40.7)	
Rhinitis diagnosis			
n (% non-missing)	27 (100.0)	27 (100.0)	0.1628
Yes	8 (29.6)	13 (48.1)	
IHD diagnosis			
n (% non-missing)	27 (100.0)	27 (100.0)	0.5525
Yes	2 (7.4)	1 (3.7)	
Heart failure diagnosis			
n (% non-missing)	27 (100.0)	27 (100.0)	
Yes	0 (0.0)	0 (0.0)	
Hypertension diagnosis			
n (% non-missing)	27 (100.0)	27 (100.0)	0.4436
Yes	5 (18.5)	3 (11.1)	
Diabetes diagnosis			
n (% non-missing)	27 (100.0)	27 (100.0)	
Yes	0 (0.0)	0 (0.0)	
GERD active diagnosis			
n (% non-missing)	27 (100.0)	27 (100.0)	0.3128
Yes	1 (3.7)	0 (0.0)	
Predicted peak flow			
n (% non-missing)	14 (51.9)	20 (74.1)	0.9721
Mean (SD)	488.9 (53.4)	505.8 (74.9)	
Median (IQR)	482.7 (47.2)	478.3 (127.7)	
ICS/LABA prescriptions per patient			
n (% non-missing)	27 (100.0)	27 (100.0)	0.6546
Mean (SD)	3.6 (4.3)	3.2 (2.5)	
Median (IQR)	1.0 (7.0)	3.0 (5.0)	
Mono ICS prescriptions per patient			
n (% non-missing)	27 (100.0)	27 (100.0)	0.4898
Mean (SD)	1.2 (1.9)	1.0 (2.2)	
Median (IQR)	0.0 (2.0)	0.0 (1.0)	
Mean daily SABA dosage (µg)			
n (% non-missing)	27 (100.0)	27 (100.0)	0.5066
< 100	8 (29.6)	6 (22.2)	
100–200	11 (40.7)	8 (29.6)	
201–400	6 (22.2)	8 (29.6)	
> 400	2 (7.4)	5 (18.5)	
ICS adherence^b^			
n (% non-missing)	27 (100.0)	27 (100.0)	0.7158
Mean (SD)	57.1 (39.3)	52.3 (32.9)	
Median (IQR)	54.8 (54.8)	49.3 (35.7)	

All values in the table are n (%) unless otherwise specified. High FeNO defined as ≥ 50 ppb; non-high FeNO < 25 ppb; high blood eosinophil count defined as ≥ 0.300 × 10^9^ cells/L; non-high blood eosinophil count < 0.300 × 10^9^ cells/L

*ATS* American Thoracic Society, *BMI* body mass index, *FeNO* fractional exhaled nitric oxide, *GERD* gastroesophageal reflux disease, *ICS* inhaled corticosteroid, *IHD* ischaemic heart disease, *IQR* interquartile range, *LABA* long-acting β_2_-agonist, *ppb* parts per billion, *SABA* short-acting β_2_-agonist, *SD* standard deviation

^a^Active denotes diagnosed in the year before FeNO reading or treated in the year before FeNO reading

^b^Medication Possession Ratio was calculated by dividing the total of 1 day’s supply by the total number of days evaluated, multiplied by 100%. The evaluation period for all patients was 365 days in the study year

**Table 2:** Categorisation 1: non-high FeNO and non-high blood eosinophils vs. high FeNO or high blood eosinophils

Characteristics	Non-high FeNO and non-high blood eosinophils (n = 200)	High FeNO or high blood eosinophils (n = 200)	p-value
Sex			
n (% non-missing)	200 (100.0)	200 (100.0)	1.0000
Male	84 (42.0)	84 (42.0)	
Age			
n (% non-missing)	200 (100.0)	200 (100.0)	0.9223
Mean (SD)	51.7 (13.1)	51.6 (13.2)	
Median (IQR)	54.0 (18.5)	53.0 (19.5)	
Age group			
n (% non-missing)	200 (100.0)	200 (100.0)	0.4289
Under 35	24 (12.0)	25 (12.5)	
35–65	150 (75.0)	140 (70.0)	
66–80	26 (13.0)	35 (17.5)	
Smoking status			
n (% non-missing)	200 (100.0)	200 (100.0)	1.0000
Non-smoker	73 (36.5)	73 (36.5)	
Ex-smoker	23 (11.5)	23 (11.5)	
Current smoker	71 (35.5)	71 (35.5)	
BMI			
n (% non-missing)	189 (94.5)	191 (95.5)	0.1025
Mean (SD)	30.0 (6.9)	29.1 (7.0)	
Median (IQR)	28.7 (8.1)	27.8 (7.9)	
Active eczema diagnosis^a^			
n (% non-missing)	200 (100.0)	200 (100.0)	0.3347
Yes	7 (3.5)	11 (5.5)	
Active rhinitis diagnosis^a^			
n (% non-missing)	200 (100.0)	200 (100.0)	0.1056
Yes	55 (27.5)	70 (35.0)	
Eczema diagnosis			
n (% non-missing)	200 (100.0)	200 (100.0)	0.1284
Yes	54 (27.0)	68 (34.0)	
Rhinitis diagnosis			
n (% non-missing)	200 (100.0)	200 (100.0)	0.0858
Yes	77 (38.5)	94 (47.0)	
IHD diagnosis			
n (% non-missing)	200 (100.0)	200 (100.0)	1.0000
Yes	9 (4.5)	9 (4.5)	
Heart failure diagnosis			
n (% non-missing)	200 (100.0)	200 (100.0)	0.3167
Yes	0 (0.0)	1 (0.5)	
Hypertension diagnosis			
n (% non-missing)	200 (100.0)	200 (100.0)	0.0592
Yes	55 (27.5)	39 (19.5)	
Diabetes diagnosis			
n (% non-missing)	200 (100.0)	200 (100.0)	0.8630
Yes	19 (9.5)	18 (9.0)	
GERD active diagnosis			
n (% non-missing)	200 (100.0)	200 (100.0)	0.0630
Yes	35 (17.5)	22 (11.0)	
Predicted peak flow			
n (% non-missing)	105 (52.5)	110 (55.0)	0.7422
Mean (SD)	516.0 (73.2)	519.4 (75.8)	
Median (IQR)	485.8 (134.7)	487.6 (137.8)	
ICS/LABA prescriptions per patient			
n (% non-missing)	200 (100.0)	200 (100.0)	0.4736
Mean (SD)	4.1 (4.0)	4.1 (3.7)	
Median (IQR)	3.0 (5.0)	3.5 (5.0)	
Mono ICS prescriptions per patient			
n (% non-missing)	200 (100.0)	200 (100.0)	0.0112
Mean (SD)	1.4 (2.7)	0.6 (1.6)	
Median (IQR)	0.0 (1.0)	0.0 (0.0)	
Mean daily SABA dosage (µg)			
n (% non-missing)	200 (100.0)	200 (100.0)	0.2808
< 100	67 (33.5)	83 (41.5)	
100–200	58 (29.0)	47 (23.5)	
201–400	45 (22.5)	47 (23.5)	
> 400	30 (15.0)	23 (11.5)	
ICS adherence^b^			
n (% non-missing)	200 (100.0)	200 (100.0)	0.1931
Mean (SD)	72.2 (72.7)	63.3 (53.3)	
Median (IQR)	61.7 (64.4)	52.0 (61.7)	

All values in table are n (%) unless otherwise specified. High FeNO defined as ≥ 50 ppb; non-high FeNO < 25 ppb; high blood eosinophil count defined as ≥ 0.300 × 10^9^ cells/L; non-high blood eosinophil count < 0.300 × 10^9^ cells/L

*ATS* American Thoracic Society, *BMI* body mass index, *FeNO* fractional exhaled nitric oxide, *GERD* gastroesophageal reflux disease, *ICS* inhaled corticosteroid, *IHD* ischaemic heart disease, *IQR* interquartile range, *LABA* long-acting β_2_-agonist, *ppb* parts per billion, *SABA* short-acting β_2_-agonist, *SD* standard deviation

^a^Active denotes diagnosed in the year before FeNO reading or treated in the year before FeNO reading

^b^Medication Possession Ratio was calculated by dividing the total of 1 day’s supply by the total number of days evaluated, multiplied by 100%. The evaluation period for all patients was 365 days in the study year

Demographics and clinical characteristics were generally similar between the matched groups. However, standalone ICS prescriptions were significantly fewer in the high FeNO or high blood eosinophil cohort compared with the reference group (0.6 vs. 1.4 mean standalone ICS prescriptions/patient, p = 0.0112). Adherence to ICS was comparable between matched groups and was 52.3% and 63.3% in the high FeNO and high blood eosinophil cohort and high FeNO or high blood eosinophil cohort, respectively.

#### Categorisation 2

Across the biomarker cohorts, more than 50% of patients were female, with most patients aged 35–65 years. Non-smokers represented 36–58.5% of the study sample.

Patients in the non-high FeNO and high blood eosinophil cohort, high FeNO and non-high blood eosinophil cohort, and high FeNO and high blood eosinophil cohort had significantly lower BMI compared with the reference group (29.0 vs. 30.1 kg/m^2^, p = 0.0492; 26.9 vs. 29.3 kg/m^2^, p = 0.0063; and 26.8 vs. 29.0 kg/m^2^, p = 0.0386, respectively). All other baseline demographics were well-balanced between the matched groups (Tables 3, 4, 5). For comorbidities, a greater number of patients had a diagnosis of rhinitis in the non-high FeNO and high blood eosinophil cohort compared with the reference group (88 vs. 67 patients, p = 0.0272). In addition, differences were observed in the number of ICS prescriptions per patient. Patients in the non-high FeNO and high blood eosinophil cohort, as well as the high FeNO and non-high blood eosinophil cohort, had fewer standalone ICS prescriptions per patient relative to the reference group (0.7 vs. 1.3 mean standalone ICS prescriptions/patient, p = 0.0362 and 0.9 vs. 1.6, p = 0.0295, respectively). Adherence to ICS was not significantly different between matched groups and was 66.2%, 65.7%, and 68.6% in the non-high FeNO and high blood eosinophil, high FeNO and non-high blood eosinophil, and high FeNO and high blood eosinophil cohorts, respectively.

**Table 3:** Categorisation 2: non-high FeNO and non-high blood eosinophils vs. non-high FeNO and high blood eosinophils

Characteristics	Non-high FeNO and non-high blood eosinophils (n = 186)	Non-high FeNO and high blood eosinophils (n = 186)	p-value
Sex			
n (% non-missing)	186 (100.0)	186 (100.0)	1.0000
Male	77 (41.4)	77 (41.4)	
Age			
n (% non-missing)	186 (100.0)	186 (100.0)	0.9834
Mean (SD)	51.9 (13.1)	51.8 (13.7)	
Median (IQR)	55.0 (20.0)	53.5 (20.0)	
Age group			
n (% non-missing)	186 (100.0)	186 (100.0)	0.1919
Under 35	22 (11.8)	24 (12.9)	
35–65	141 (75.8)	127 (68.3)	
44–80	23 (12.4)	35 (18.8)	
Smoking status			
n (% non-missing)	186 (100.0)	186 (100.0)	1.0000
Non-smoker	67 (36.0)	67 (36.0)	
Ex-smoker	22 (11.8)	22 (11.8)	
Current smoker	67 (36.0)	67 (36.0)	
BMI			
n (% non-missing)	184 (98.4)	184 (98.4)	0.0492
Mean (SD)	30.1 (6.3)	29.0 (6.7)	
FeNO			
n (% non-missing)	186 (100.0)	186 (100.0)	<0.0001
Mean (SD)	16.5 (7.8)	28.9 (23.8)	
Median (IQR)	16.0 (12.0)	23.0 (22.0)	
Blood eosinophil count			
n (% non-missing)	186 (100.0)	184 (98.9)	<0.0001
Mean (SD)	0.2 (0.1)	0.4 (0.2)	
Median (IQR)	0.2 (0.1)	0.4 (0.2)	
Active eczema diagnosis^a^			
n (% non-missing)	186 (100.0)	186 (100.0)	0.1876
Yes	5 (2.7)	10 (5.4)	
Active rhinitis diagnosis^a^			
n (% non-missing)	186 (100.0)	186 (100.0)	0.0720
Yes	49 (26.3)	65 (34.9)	
Eczema diagnosis			
n (% non-missing)	186 (100.0)	186 (100.0)	0.2273
Yes	57 (30.6)	68 (36.6)	
Rhinitis diagnosis			
n (% non-missing)	186 (100.0)	186 (100.0)	0.0272
Yes	67 (36.0)	88 (47.3)	
IHD diagnosis			
n (% non-missing)	186 (100.0)	186 (100.0)	0.4564
Yes	7 (3.8)	10 (5.4)	
Heart failure diagnosis			
n (% non-missing)	186 (100.0)	186 (100.0)	0.3167
Yes	0 (0.0)	1 (0.5)	
Hypertension diagnosis			
n (% non-missing)	186 (100.0)	186 (100.0)	0.3212
Yes	46 (24.7)	38 (20.4)	
Diabetes diagnosis			
n (% non-missing)	186 (100.0)	186 (100.0)	0.5736
Yes	14 (7.5)	17 (9.1)	
GERD active diagnosis			
N (% non-missing)	186 (100.0)	186 (100.0)	0.4352
Yes	26 (14.0)	21 (11.3)	
Predicted peak flow			
n (% non-missing)	98 (52.7)	101 (54.3)	0.8525
Mean (SD)	515.7 (75.3)	517.3 (76.2)	
ICS/LABA prescriptions per patient			
n (% non-missing)	186 (100.0)	186 (100.0)	0.0404
Mean (SD)	3.8 (3.9)	4.4 (3.8)	
Median (IQR)	3.0 (6.0)	4.0 (5.0)	
Mono ICS prescriptions per patient			
n (% non-missing)	186 (100.0)	186 (100.0)	0.0362
Mean (SD)	1.3 (2.5)	0.7 (1.6)	
Median (IQR)	0.0 (1.0)	0.0 (1.0)	
Mean daily SABA dosage (µg)			
n (% non-missing)	186 (100.0)	186 (100.0)	0.2585
<100	63 (33.9)	71 (38.2)	
100–200	57 (30.6)	48 (25.8)	
201–400	35 (18.8)	45 (24.2)	
>400	31 (16.7)	22 (11.8)	
ICS adherence^b^			
n (% non-missing)	186 (100.0)	186 (100.0)	0.8806
Mean (SD)	69.2 (55.2)	66.2 (53.3)	
Median (IQR)	54.8 (71.2)	56.1 (57.5)	

All values in the table are n (%) unless otherwise specified. High FeNO defined as ≥ 35 ppb; non-high FeNO < 35 ppb; high blood eosinophil count defined as ≥ 0.300 × 10^9^ cells/L; non-high blood eosinophil count < 0.300 × 10^9^ cells/L

*BMI* body mass index, *FeNO* fractional exhaled nitric oxide, *GERD* gastroesophageal reflux disease, *ICS* inhaled corticosteroid, *IHD* ischaemic heart disease, *IQR* interquartile range, *LABA* long-acting β_2_-agonist, *ppb* parts per billion, *SABA* short-acting β_2_-agonist, SD standard deviation

^a^Active denotes diagnosed in the year before FeNO reading or treated in the year before FeNO reading

^b^Medication Possession Ratio was calculated by dividing the total of 1 day’s supply by the total number of days evaluated, multiplied by 100%. The evaluation period for all patients was 365 days in the study year

**Table 4:** Categorisation 2: non-high FeNO and non-high blood eosinophils vs. high FeNO and non-high blood eosinophils

Characteristics	Non-high FeNO and non-high blood eosinophils (n = 98)	High FeNO and non-high blood eosinophils (n = 98)	p-value
Sex			
n (% non-missing)	98 (100.0)	98 (100.0)	1.0000
Male	41 (41.8)	41 (41.8)	
Age			
n (% non-missing)	98 (100.0)	98 (100.0)	1.0000
Mean (SD)	48.8 (15.3)	48.6 (15.6)	
Median (IQR)	53.0 (27.0)	53.0 (27.0)	
Age group			
n (% non-missing)	98 (100.0)	98 (100.0)	0.3072
Under 35	23 (23.5)	24 (24.5)	
35–65	65 (66.3)	57 (58.2)	
66–80	10 (10.2)	17 (17.3)	
Smoking status			
n (% non-missing)	98 (100.0)	98 (100.0)	1.0000
Non-smoker	53 (54.1)	53 (54.1)	
Ex-smoker	8 (8.2)	8 (8.2)	
Current smoker	23 (23.5)	23 (23.5)	
BMI			
n (% non-missing)	96 (98.0)	94 (95.9)	0.0063
Mean (SD)	29.3 (6.2)	26.9 (5.8)	
Median (IQR)	27.9 (8.6)	25.7 (7.6)	
FeNO			
n (% non-missing)	98 (100.0)	98 (100.0)	< 0.0001
Mean (SD)	17.7 (7.9)	60.0 (31.8)	
Median (IQR)	17.0 (10.0)	50.0 (25.0)	
Blood eosinophil count			
n (% non-missing)	98 (100.0)	97 (99.0)	< 0.0001
Mean (SD)	0.2 (0.1)	0.3 (0.3)	
Median (IQR)	0.2 (0.1)	0.3 (0.3)	
Active eczema diagnosis^a^			
n (% non-missing)	98 (100.0)	98 (100.0)	1.0000
Yes	4 (4.1)	4 (4.1)	
Active rhinitis diagnosis^a^			
n (% non-missing)	98 (100.0)	98 (100.0)	0.7492
Yes	26 (26.5)	28 (28.6)	
Eczema diagnosis			
n (% non-missing)	98 (100.0)	98 (100.0)	0.8763
Yes	29 (29.6)	30 (30.6)	
Rhinitis diagnosis			
n (% non-missing)	98 (100.0)	98 (100.0)	0.4546
Yes	32 (32.7)	37 (37.8)	
IHD diagnosis			
n (% non-missing)	98 (100.0)	98 (100.0)	0.7003
Yes	4 (4.1)	3 (3.1)	
Heart failure diagnosis			
n (% non-missing)	98 (100.0)	98 (100.0)	
Yes	0 (0.0)	0 (0.0)	
Hypertension diagnosis			
n (% non-missing)	98 (100.0)	98 (100.0)	0.0967
Yes	29 (29.6)	19 (19.4)	
Diabetes diagnosis			
n (% non-missing)	98 (100.0)	98 (100.0)	0.5513
Yes	7 (7.1)	5 (5.1)	
GERD active diagnosis			
n (% non-missing)	98 (100.0)	98 (100.0)	0.6018
Yes	9 (9.2)	7 (7.1)	
Predicted peak flow			
n (% non-missing)	55 (56.1)	71 (72.4)	0.6615
Mean (SD)	520.7 (67.4)	515.9 (68.6)	
Median (IQR)	490.8 (137.4)	493.7 (125.0)	
ICS/LABA prescriptions per patient			
n (% non-missing)	98 (100.0)	98 (100.0)	0.1318
Mean (SD)	2.6 (3.1)	3.4 (3.7)	
Median (IQR)	1.0 (4.0)	2.0 (5.0)	
Mono ICS prescriptions per patient			
n (% non-missing)	98 (100.0)	98 (100.0)	0.0295
Mean (SD)	1.6 (2.8)	0.9 (1.9)	
Median (IQR)	0.0 (2.0)	0.0 (1.0)	
Mean daily SABA dosage (µg)			
n (% non-missing)	98 (100.0)	98 (100.0)	0.3731
< 100	31 (31.6)	40 (40.8)	
100–200	32 (32.7)	22 (22.4)	
201–400	20 (20.4)	22 (22.4)	
> 400	15 (15.3)	14 (14.3)	
ICS adherence^b^			
n (% non-missing)	98 (100.0)	98 (100.0)	0.4778
Mean (SD)	58.1 (43.5)	65.7 (67.6)	
Median (IQR)	49.3 (57.5)	49.3 (54.8)	

All values in the table are n (%) unless otherwise specified. High FeNO defined as ≥ 35 ppb; non-high FeNO < 35 ppb; high blood eosinophil count defined as ≥ 0.300 × 10^9^ cells/L; non-high blood eosinophil count < 0.300 × 10^9^ cells/L

*BMI* body mass index, *FeNO* fractional exhaled nitric oxide, *GERD* gastroesophageal reflux disease, *ICS* inhaled corticosteroid, *IHD* ischaemic heart disease, *IQR* interquartile range, *LABA* long-acting β_2_-agonist, *ppb* parts per billion, *SABA* short-acting β_2_-agonist, *SD* standard deviation

^a^Active denotes diagnosed in the year before FeNO reading or treated in the year before FeNO reading

^b^Medication Possession Ratio was calculated by dividing the total of 1 day’s supply by the total number of days evaluated, multiplied by 100%. The evaluation period for all patients was 365 days in the study year

**Table 5:** Categorisation 2: non-high FeNO and non-high blood eosinophils vs. high FeNO and high blood eosinophils

Characteristics	Non-high FeNO and non-high blood eosinophils (n = 53)	High FeNO and high blood eosinophils (n = 53)	p-value
Sex			
n (% non-missing)	53 (100.0)	53 (100.0)	1.0000
Male	48.4 (16.7)	48.2 (16.9)	
Age			
n (% non-missing)	53.0 (100.0)	53.0 (100.0)	0.9647
Mean (SD)	48.4 (16.7)	48.2 (16.9)	
Median (IQR)	53.0 (32.0)	53.0 (31.0)	
Age group			
n (% non-missing)	53 (100.0)	53 (100.0)	0.8655
Under 35	14 (26.4)	14 (26.4)	
35–65	31 (58.5)	29 (54.7)	
66–80	8 (15.1)	10 (18.9)	
Smoking status			
n (% non-missing)	46 (86.8)	46 (86.8)	1.0000
Non-smoker	31 (58.5)	31 (58.5)	
Ex-smoker	4 (7.5)	4 (7.5)	
Current smoker	11 (20.8)	11 (20.8)	
BMI			
n (% non-missing)	52 (98.1)	51 (96.2)	0.0386
Mean (SD)	29.0 (5.9)	26.8 (5.6)	
Median (IQR)	28.4 (8.3)	25.6 (7.1)	
FeNO			
n (% non-missing)	53 (100.0)	53 (100.0)	< 0.0001
Mean (SD)	18.6 (7.7)	57.8 (26.4)	
Median (IQR)	20.0 (10.0)	49.0 (28.0)	
Blood eosinophil count			
n (% non-missing)	53 (100.0)	52 (98.1)	< 0.0001
Mean (SD)	0.1 (0.1)	0.5 (0.3)	
Median (IQR)	0.1 (0.1)	0.5 (0.2)	
Active eczema diagnosis^a^			
n (% non-missing)	53 (100.0)	53 (100.0)	1.0000
Yes	2 (3.8)	2 (3.8)	
Active rhinitis diagnosis^a^			
n (% non-missing)	53 (100.0)	53 (100.0)	0.4052
Yes	15 (28.3)	19 (35.8)	
Eczema diagnosis			
n (% non-missing)	53 (100.0)	53 (100.0)	0.5355
Yes	16 (30.2)	19 (35.8)	
Rhinitis diagnosis			
n (% non-missing)	53 (100.0)	53 (100.0)	0.0451
Yes	15 (28.3)	25 (47.2)	
IHD diagnosis			
n (% non-missing)	53 (100.0)	53 (100.0)	0.5581
Yes	1 (1.9)	2 (3.8)	
Heart failure diagnosis			
n (% non-missing)	53 (100.0)	53 (100.0)	
Yes	0 (0.0)	0 (0.0)	
Hypertension diagnosis			
n (% non-missing)	53 (100.0)	53 (100.0)	0.0990
Yes	15 (28.3)	8 (15.1)	
Diabetes diagnosis			
n (% non-missing)	53 (100.0)	53 (100.0)	1.0000
Yes	3 (5.7)	3 (5.7)	
GERD active diagnosis			
n (% non-missing)	53 (100.0)	53 (100.0)	0.5063
Yes	6 (11.3)	4 (7.5)	
Predicted peak flow			
n (% non-missing)	35 (66.0)	39 (73.6)	0.8838
Mean (SD)	513.0 (66.7)	510.3 (69.9)	
Median (IQR)	487.5 (122.0)	481.2 (128.0)	
ICS/LABA prescriptions per patient			
n (% non-missing)	53 (100.0)	53 (100.0)	0.2204
Mean (SD)	3.3 (3.9)	4.0 (4.0)	
Median (IQR)	2.0 (5.0)	3.0 (5.0)	
Mono ICS prescriptions per patient			
n (% non-missing)	53 (100.0)	53 (100.0)	0.1944
Mean (SD)	1.3 (2.3)	0.8 (1.7)	
Median (IQR)	0.0 (1.0)	0.0 (1.0)	
Mean daily SABA dosage (µg)			
n (% non-missing)	53 (100.0)	53 (100.0)	0.6923
< 100	15 (28.3)	16 (30.2)	
100–200	19 (35.8)	14 (26.4)	
201–400	10 (18.9)	14 (26.4)	
> 400	9 (17.0)	9 (17.0)	
ICS adherence^b^			
n (% non-missing)	53 (100.0)	53 (100.0)	0.8149
Mean (SD)	64.1 (45.3)	68.6 (72.5)	
Median (IQR)	54.8 (54.8)	49.3 (49.3)	

All values in the table are n (%) unless otherwise specified. High FeNO defined as ≥ 35 ppb; non-high FeNO < 35 ppb; high blood eosinophil count defined as ≥ 0.300 × 10^9^ cells/L; non-high blood eosinophil count < 0.300 × 10^9^ cells/L

*BMI* body mass index, *FeNO* fractional exhaled nitric oxide, *GERD* gastroesophageal reflux disease, *ICS* inhaled corticosteroid, *IHD* ischaemic heart disease, *IQR* interquartile range, *LABA* long-acting β_2_-agonist, *ppb* parts per billion, *SABA* short-acting β_2_-agonist, *SD* standard deviation

^a^Active denotes diagnosed in the year before FeNO reading or treated in the year before FeNO reading

^b^Medication Possession Ratio was calculated by dividing the total of 1 day’s supply by the total number of days evaluated, multiplied by 100%. The evaluation period for all patients was 365 days in the study year

### Asthma Exacerbations

#### Categorisation 1

**Fig. 3:**
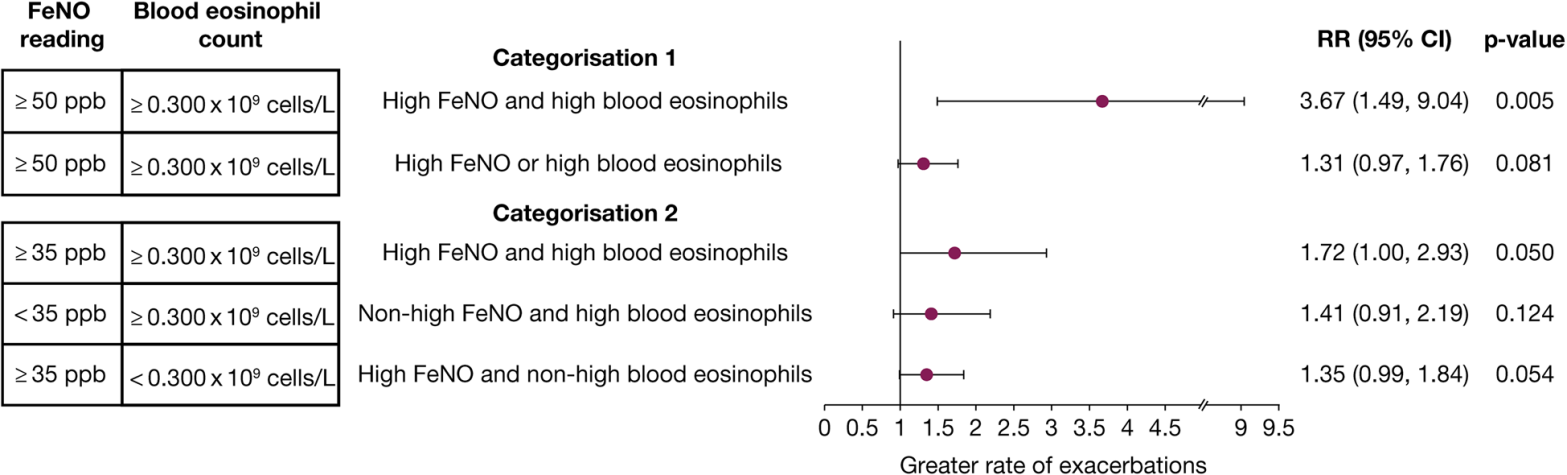
Comparison of exacerbation rates of cohorts relative to non-high FeNO and non-high blood eosinophil cohorts. *CI* confidence interval, *FeNO* fractional exhaled nitric oxide, *RR* rate ratio

In the high FeNO and high blood eosinophil count cohort, a significantly greater percentage of patients were in the greater exacerbation categories compared with patients in the reference group (p = 0.0427) (Additional file 1: Table S3). The mean (SD) number of exacerbations was also significantly greater relative to the reference group (0.8 [1.0] vs. 0.2 [0.4]; p = 0.0109). Overall, the estimated rate of exacerbations in the high FeNO and high blood eosinophil cohort was statistically significantly greater (unadjusted RR: 3.67 [95% CI: 1.49, 9.04], p = 0.005) compared with matched patients in the reference group (Fig. 3). Likewise, significantly more patients were in the greater exacerbation categories in the high FeNO or high blood eosinophil cohort compared with patients in the reference group (p = 0.0481); however, the mean (SD) number of exacerbations was not significantly different from that in the reference group (0.5 [0.8] vs. 0.4 [0.6]; p = 0.3423) (Additional file 1: Table S3). Overall, the exacerbation rate was numerically greater but did not reach statistical significance when compared with matched patients in the reference group (1.31 [95% CI: 0.97, 1.76], p = 0.081).

#### Categorisation 2

In both the non-high FeNO and high blood eosinophil cohort and the high FeNO and non-high blood eosinophil cohort, the mean number of exacerbations was not significantly different from those for the reference groups (0.5 [0.9] vs. 0.4 [0.7], p = 0.3134 and 0.5 [0.7] vs. 0.3 [0.6], p = 0.1332, respectively) (Additional file 1: Table S3). While both groups demonstrated a clear trend towards greater exacerbation rates (1.41 [95% CI: 0.91, 2.19], p = 0.124 and 1.35 [95% CI: 0.99, 1.84], p = 0.054, respectively) in comparison with the reference group, this did not reach statistical significance (Fig. 3). For the high FeNO and high blood eosinophil cohort (both biomarkers elevated), although the mean number of exacerbations was not significantly different from that in the reference group (0.7 [0.9] vs. 0.4 [0.7], p = 0.116) (Additional file 1: Table S3), a significantly greater exacerbation rate was observed (1.72 [95% CI: 1.00, 2.93], p = 0.050) compared with the reference group (Fig. 3).

## Discussion

With the development of new biologics that target eosinophilic airway inflammation, accurate and easy-to-use biomarkers to predict asthma exacerbations and likely patient responses to treatment are needed. We conducted a real-world matched cohort study to investigate the relationship between blood eosinophil count, FeNO readings, and the severe exacerbation rate observed in asthma patients prescribed ICS.

We observed that for categorisation 1, based on ATS criteria for FeNO cutoffs, patients with a high FeNO (≥ 50 ppb) and high blood eosinophil count (≥ 0.300 × 10^9^ cells/L) were almost four-times as likely to have had a severe exacerbation compared with patients with non-high FeNO (< 25 ppb) and non-high blood eosinophil count (< 0.300 × 10^9^ cells/L) in the year preceding the FeNO reading. In patients with either a high FeNO reading or a high blood eosinophil count, the exacerbation RR was less pronounced and non-significant compared with the reference group. In categorisation 2, patients in the high FeNO (> 35 ppb) and high eosinophil count (≥ 0.300 × 10^9^ cells/L) cohort were almost twice as likely to have severe exacerbations in the year prior to the FeNO reading compared with the reference group, whereas the high FeNO and non-high blood eosinophil count cohort and non-high FeNO and high blood eosinophil count cohort displayed a trend towards increased exacerbations relative to the reference group, that did not reach statistical significance. Therefore, the combination of blood eosinophil count and FeNO may be an even stronger marker of exacerbation risk compared with the individual biomarkers. Moreover, the use of the ATS criteria for high FeNO (≥ 50 ppb) resulted in a greater estimated exacerbation rate, indicating that a greater FeNO reading (≥ 50 ppb vs. ≥ 35 ppb) in the presence of a raised blood eosinophil count was associated with an even greater exacerbation rate. Notably, the exacerbation risk seemed to be independent of traditionally used prognostic variables such as predicted peak flow and short-acting β_2_-agonist use, which were not significantly different between cohorts.

The cutoffs used in the study to define high FeNO concentration and high blood eosinophil count warrant further consideration. The cutoff chosen for high blood eosinophil count (≥ 0.300 × 10^9^ cells/L) was well within the range of peripheral blood eosinophils (usually ranging between 0.200 × 10^9^ cells/L and 0.300 × 10^9^ cells/L) that most accurately predicts sputum eosinophil count in patients with severe asthma [36]. For FeNO classification, the ATS criteria for adults is commonly used, wherein the high FeNO cutoff has been set at > 50 ppb and low FeNO at < 25 ppb [19, 23, 37]. As cutoff concentrations for high, medium, and low FeNO may be confusing for clinicians with relatively little experience of FeNO as a biomarker, we tested a simplified FeNO cutoff criteria (high FeNO, ≥ 35 ppb; non-high FeNO, < 35 ppb) for ease of use in primary care settings. The high FeNO cutoff of ≥ 35 ppb has also been validated in several studies, in turn, identifying patients with uncontrolled asthma and a more severe asthma phenotype [31, 32]. These results suggest that a lower high FeNO cutoff of ≥ 35 ppb instead of ≥ 50 ppb (ATS criteria), on a background of raised blood eosinophil count, may still be relevant to predict those patients at significant risk of severe exacerbations. This implies that asthma patients with comparatively lower raised FeNO concentrations and elevated blood eosinophil count may require further treatment, suggesting that the risk of severe exacerbations may potentially be over and above that provided by a traditional severity-based classification.

Few studies have evaluated the predictive value of the combination of blood eosinophil count and FeNO concentration in asthma. However, available studies have demonstrated that combining FeNO and blood eosinophil count has an additive effect in predicting wheeze, frequent exacerbations, impaired lung function, and bronchial hyper-responsiveness [23, 38]. The National Institute for Health and Care Excellence [39] and the British Thoracic Society recommend FeNO measurement to guide diagnosis and treatment of eosinophilic asthma [40]. Use of FeNO as a diagnostic tool is increasing. In UK primary care practices, FeNO monitoring is also being used to guide decisions on ICS usage or step-up therapy [37]. In addition, the 2019 Global Initiative for Asthma strategy report [14] recommends the use of FeNO and/or blood eosinophil counts to determine asthma phenotype and for biomarker-guided selection of biologics. Thus, composite, non-invasive biomarkers, such as FeNO and easily obtainable blood eosinophil count, may provide insight into a patient’s risk of exacerbations as well as guide asthma treatment.

Other well-characterised risk factors for asthma exacerbations include prior exacerbations, OCS use, and underlying lung function impairment [41, 42]. The combination of these standard medical history/lung function-based assessments and objective biomarkers, such as FeNO and blood eosinophil count, may improve the prediction of asthma exacerbations. Furthermore, within the limits of the data, our results indicate that the prognostic value of both FeNO and blood eosinophil count as complementary biomarkers appears to be greater than that provided by these traditional clinical assessments [41, 42].

This study has several limitations. The power analysis performed at the protocol stage demonstrated that more patients were required for sufficient power to demonstrate a difference between four groups than were available. Secondly, the OPCRD data set comprised information collected for clinical and routine use rather than specifically for research purposes. Although extensive quality control and validity checks were conducted at the practice level, the validity and completeness of individual patient records can be limited. Since blood eosinophil measurements and FeNO readings are not collected routinely, patients with asthma who had both blood eosinophil counts and FeNO measured may not have been representative of the overall asthma population. In addition, the time from when the blood eosinophil count reading was taken to the index date varied considerably. Although high blood eosinophil counts have been observed to be a stable phenotype, at least during a 1-year period [11], further studies are required to investigate the potential long-term stability of blood eosinophil counts. As with all observational studies, confounding variables, arising from systematic differences between the patients being compared, may have complicated interpretation of these results. In this study, confounding was minimised by fitting multivariate models that adjusted patient characteristics that may have varied between patient groups. However, despite these measures, confounding by unmeasured variables may have been present. Finally, adherence to ICS was not a prerequisite to enter the study, and as a result adherence was not optimal. While ICS adherence between each cohort and reference group was not significantly different, it is likely that FeNO concentrations and blood eosinophil counts may be differentially predictive in patients receiving or not receiving their prescribed ICS medications.

Results of this study need to be confirmed in a prospective study in a larger patient population before high FeNO concentrations and high blood eosinophil counts can be advocated as a composite biomarker. Notably, patients with elevated FeNO concentration on a background of high blood eosinophil counts represent a potentially high-risk group of patients. Such severe asthma patients will benefit from studies conducted in larger epidemiological cohorts in primary care settings, as well as in severe asthma cohorts, such as the International Severe Asthma Registry [43], a global registry of adult patients with severe asthma, and the CHRONICLE study [44], an ongoing non-interventional, prospective cohort study of adults with severe asthma treated by specialists in the United States. Overall, findings from this study, based on real-life data obtained from a validated database, warrant further investigation into the role of FeNO and blood eosinophils as biomarkers in the treatment and management of asthma.

## Conclusions

The combination of raised FeNO concentrations and raised blood eosinophil counts was associated with a greater exacerbation rate compared with neither biomarker raised in the year preceding the FeNO reading. FeNO concentration and blood eosinophil count are simple measurements that could, together, improve the identification of patients with asthma in primary and secondary care at risk of exacerbations, and thus, guide additional considerations in the treatment of their asthma.

## Additional file

**Additional file 1.** Statistically significant differences between unmatched patient groups for categorisation 1 and 2 and frequency of exacerbations between matched biomarker groups.

## Data Availability

Data underlying the findings described in this manuscript may be obtained in accordance with AstraZeneca’s data sharing policy described at https://astrazenecagrouptrials.pharmacm.com/ST/Submission/Disclosure.

## Abbreviations

ADEPT: Anonymised Data Ethics and Protocol Transparency
ATS: American Thoracic Society
BEC: blood eosinophil count
BMI: body mass index
CI: confidence interval
FeNO: fractional exhaled nitric oxide
ICS: inhaled corticosteroid
OCS: oral corticosteroids
OPCRD: optimum patient care research database
RR: rate ratio
SD: standard deviation
