# Supplementary material for: Association of elevated fractional exhaled nitric oxide concentration and blood eosinophil count with severe asthma exacerbations
Source: Clin Transl Allergy. 2019 Aug 21;9:41. doi: 10.1186/s13601-019-0282-7 (PMC6702739; doi:10.1186/s13601-019-0282-7)
Supplement: Supplementary file 1 — Additional file 1. Statistically significant differences between unmatched patient groups for categorisation 1 and 2 and frequency of exacerbations between matched biomarker groups. [file 13601_2019_282_MOESM1_ESM.docx]

Additional file 1: Table S1. Statistically significant differences between unmatched patient groups (categorisation 1)

| **Characteristics** | | **Non-high blood eosinophil and low FeNO** | **Non-high blood eosinophil and medium FeNO** | **Non-high blood eosinophil and high FeNO** | **High blood eosinophil and low FeNO** | **High blood eosinophil and medium FeNO** | **High blood eosinophil and high FeNO** | **p-value** |
| --- | --- | --- | --- | --- | --- | --- | --- | --- |
| **Categorised age group** | n (% non-missing) | 296 (100.0) | 72 (100.0) | 26 (100.0) | 122 (100.0) | 67 (100.0) | 27 (100.0) | <0.0001 |
|  | Under 35 | 28 (9.5) | 12 (16.7) | 8 (30.8) | 12 (9.8) | 6 (9.0) | 12 (44.4) |  |
|  | 35–65 | 229 (77.4) | 49 (68.1) | 14 (53.8) | 90 (73.8) | 48 (71.6) | 9 (33.3) |  |
|  | 66–80 | 39 (13.2) | 11 (15.3) | 4 (15.4) | 20 (16.4) | 13 (19.4) | 6 (22.2) |  |
| **Sex** | n (% non-missing | 296 (100.0) | 72 (100.0) | 26 (100.0) | 122 (100.0) | 67 (100.0) | 27 (100.0) | 0.0028 |
|  | Male | 93 (31.4) | 25 (34.7) | 14 (53.8) | 52 (42.6) | 37 (55.2) | 10 (37.0) |  |
| **Smoking status** | n (% non-missing) | 245 (82.8) | 62 (86.1) | 22 (84.6) | 101 (82.8) | 60 (89.6) | 22 (81.5) | 0.0007 |
|  | Non-smoker | 110 (44.9) | 36 (58.1) | 15 (68.2) | 30 (29.7) | 32 (53.3) | 14 (63.6) |  |
|  | Ex-smoker | 35 (14.3) | 7 (11.3) | 3 (13.6) | 29 (28.7) | 6 (10.0) | 2 (9.1) |  |
|  | Current smoker | 100 (40.8) | 19 (30.6) | 4 (18.2) | 42 (41.6) | 22 (36.7) | 6 (27.3) |  |
| **BMI** | n (% non-missing) | 280 (94.6) | 70 (97.2) | 24 (92.3) | 118 (96.7) | 63 (94.0) | 26 (96.3) | 0.0022 |
|  | Mean (SD) | 30.0 (6.7) | 29.5 (6.1) | 26.3 (5.3) | 30.8 (7.6) | 28.2 (7.1) | 26.9 (6.4) |  |
| **Eczema diagnosis** | n (% non-missing) | 296 (100.0) | 72 (100.0) | 26 (100.0) | 122 (100.0) | 67 (100.0) | 27 (100.0) | 0.0405 |
|  | Yes | 78 (26.4) | 23 (31.9) | 5 (19.2) | 49 (40.2) | 17 (25.4) | 11 (40.7) |  |
| **Rhinitis diagnosis** | n (% non-missing) | 296 (100.0) | 72 (100.0) | 26 (100.0) | 122 (100.0) | 67 (100.0) | 27 (100.0) | 0.0270 |
|  | Yes | 114 (38.5) | 24 (33.3) | 5 (19.2) | 52 (42.6) | 36 (53.7) | 13 (48.1) |  |
| **GERD active diagnosis** | n (% non-missing) | 296 (100.0) | 72 (100.0) | 26 (100.0) | 122 (100.0) | 67 (100.0) | 27 (100.0) | 0.0223 |
|  | Yes | 50 (16.9) | 5 (6.9) | 1 (3.8) | 13 (10.7) | 10 (14.9) | 0 (0.0) |  |
| **Current peak flow** | n (% non-missing) | 160 (54.1) | 49 (68.1) | 19 (73.1) | 57 (46.7) | 42 (62.7) | 20 (74.1) | 0.0285 |
|  | Mean (SD) | 401.3 (102.9) | 403.6 (112.7) | 431.1 (123.3) | 423.6 (132.1) | 469.8 (111.2) | 406.0 (142.4) |  |
| **Predicted peak flow** | n (% non-missing) | 158 (53.4) | 49 (68.1) | 19 (73.1) | 57 (46.7) | 42 (62.7) | 20 (74.1) | 0.0032 |
|  | Mean (SD) | 495.7 (71.4) | 503.2 (79.3) | 542.6 (60.1) | 526.8 (81.3) | 534.8 (69.7) | 505.8 (74.9) |  |
| **FEV_1_ % predicted groups** | n (% non-missing) | 55 (18.6) | 10 (13.9) | 4 (15.4) | 28 (23.0) | 13 (19.4) | 5 (18.5) | 0.0456 |
|  | 50% or less | 12 (21.8) | 1 (10.0) | 1 (25.0) | 10 (35.7) | 9 (69.2) | 1 (20.0) |  |
|  | >50% to <80% | 13 (23.6) | 4 (40.0) | 1 (25.0) | 8 (28.6) | 0 (0.0) | 1 (20.0) |  |
|  | 80% to <100% | 15 (27.3) | 4 (40.0) | 2 (50.0) | 3 (10.7) | 2 (15.4) | 3 (60.0) |  |
|  | 100%+ | 15 (27.3) | 1 (10.0) | 0 (0.0) | 7 (25.0) | 2 (15.4) | 0 (0.0) |  |
| **Asthma consultations** | n (% non-missing) | 296 (100.0) | 72 (100.0) | 26 (100.0) | 122 (100.0) | 67 (100.0) | 27 (100.0) | 0.0446 |
|  | Mean (SD) | 1.8 (1.5) | 2.3 (1.8) | 2.5 (2.6) | 1.7 (1.3) | 1.9 (2.0) | 2.3 (1.4) |  |
| **Asthma related unplanned inpatient attendance** | n (% non-missing) | 296 (100.0) | 72 (100.0) | 26 (100.0) | 122 (100.0) | 67 (100.0) | 27 (100.0) | 0.0163 |
|  | Yes | 0 (0.0) | 0 (0.0) | 0 (0.0) | 0 (0.0) | 1 (1.5) | 1 (3.7) |  |
| **ICS/LABA prescriptions per patient** | n (% non-missing) | 296 (100.0) | 72 (100.0) | 26 (100.0) | 122 (100.0) | 67 (100.0) | 27 (100.0) | 0.0096 |
|  | Mean (SD) | 4.2 (4.1) | 3.2 (3.5) | 2.5 (3.0) | 4.9 (3.9) | 4.2 (4.3) | 3.2 (2.5) |  |
| **ICS daily dose** | n (% non-missing) | 296 (100.0) | 72 (100.0) | 26 (100.0) | 122 (100.0) | 67 (100.0) | 27 (100.0) | 0.0454 |
|  | Mean (SD) | 586.9 (503.5) | 549.4 (477.0) | 357.4 (349.2) | 623.2 (497.7) | 588.3 (523.4) | 443.6 (342.1) |  |
| **ICS inhalers** | n (% non-missing) | 296 (100.0) | 72 (100.0) | 26 (100.0) | 122 (100.0) | 67 (100.0) | 27 (100.0) | 0.0165 |
|  | Mean (SD) | 7.0 (5.2) | 5.9 (3.9) | 4.3 (4.3) | 6.9 (4.6) | 6.9 (5.4) | 5.1 (2.8) |  |
| **ICS prescriptions** | n (% non-missing) | 296 (100.0) | 72 (100.0) | 26 (100.0) | 122 (100.0) | 67 (100.0) | 27 (100.0) | 0.0043 |
|  | Mean (SD) | 5.6 (3.8) | 4.5 (3.3) | 3.3 (3.1) | 5.5 (3.6) | 5.1 (3.9) | 4.4 (2.3) |  |
| **Number of ICS containing inhalers** | n (% non-missing) | 296 (100.0) | 72 (100.0) | 26 (100.0) | 122 (100.0) | 67 (100.0) | 27 (100.0) | 0.0010 |
|  | 1–3 | 90 (30.4) | 24 (33.3) | 17 (65.4) | 36 (29.5) | 23 (34.3) | 7 (25.9) |  |
|  | 4–6 | 76 (25.7) | 25 (34.7) | 4 (15.4) | 27 (22.1) | 16 (23.9) | 14 (51.9) |  |
|  | ≥7 | 130 (43.9) | 23 (31.9) | 5 (19.2) | 59 (48.4) | 28 (41.8) | 6 (22.2) |  |
| **Mean daily ICS dosage (µg)** | n (% non-missing) | 296 (100.0) | 72 (100.0) | 26 (100.0) | 122 (100.0) | 67 (100.0) | 27 (100.0) | 0.0026 |
|  | <250 | 89 (30.1) | 22 (30.6) | 16 (61.5) | 30 (24.6) | 21 (31.3) | 8 (29.6) |  |
|  | 250 to <500 | 74 (25.0) | 24 (33.3) | 4 (15.4) | 32 (26.2) | 19 (28.4) | 14 (51.9) |  |
|  | ≥500 | 133 (44.9) | 26 (36.1) | 6 (23.1) | 60 (49.2) | 27 (40.3) | 5 (18.5) |  |

All values in the table are n (%) unless otherwise specified. High FeNO defined as ≥50 ppb; medium FeNO, 25 to <50 ppb; low FeNO <25 ppb; high blood eosinophil count defined as ≥0.300×10^9^ cells/L; non-high blood eosinophil count <0.300×10^9^ cells/L.

BMI, body mass index; FeNO, fractional exhaled nitric oxide; FEV, forced expiratory volume; GERD, gastroesophageal reflux disease; ICS, inhaled corticosteroid; LABA, long-acting β_2_-agonist; ppb, parts per billion; SABA, short-acting β_2_-agonist; SD, standard deviation

### Additional file 1: Table S2. Statistically significant difference between unmatched patient groups (categorisation 2)

| **Characteristics** | | **Non-high eosinophil and non-high FeNO** | | **Non-high eosinophil and high FeNO** | **High eosinophil and non-high FeNO** | **High eosinophil and high FeNO** | **p-value** |
| --- | --- | --- | --- | --- | --- | --- | --- |
| **Sex** | n (% non-missing) | 347 (100.0) | | 47 (100.0) | 161 (100.0) | 55 (100.0) | 0.0177 |
|  | Male | 113 (32.6) | | 19 (40.4) | 74 (46.0) | 25 (45.5) |  |
| **Smoking status** | n (% non-missing) | 347 (100.0) | | 47 (100.0) | 161 (100.0) | 55 (100.0) | 0.0115 |
|  | Non-smoker | 138 (39.8) | | 23 (48.9) | 45 (28.0) | 31 (56.4) |  |
|  | Ex-smoker | 40 (11.5) | | 5 (10.6) | 32 (19.9) | 5 (9.1) |  |
|  | Current smoker | 111 (32.0) | | 12 (25.5) | 58 (36.0) | 12 (21.8) |  |
| **BMI** | n (% non-missing) | 331 (95.4) | | 43 (91.5) | 155 (96.3) | 51 (92.7) | 0.0005 |
|  | Mean (SD) | 29.9 (6.5) | | 27.8 (6.9) | 30.4 (7.8) | 26.7 (5.6) |  |
| **BMI** | n (% non-missing) | 347 (100.0) | | 47 (100.0) | 161 (100.0) | 55 (100.0) | 0.0411 |
|  | Underweight | 1 (0.3) | | 0 (0.0) | 2 (1.2) | 1 (1.8) |  |
|  | Normal | 81 (23.3) | | 19 (40.4) | 36 (22.4) | 20 (36.4) |  |
|  | Overweight | 102 (29.4) | | 8 (17.0) | 48 (29.8) | 18 (32.7) |  |
|  | Obese | 147 (42.4) | | 16 (34.0) | 69 (42.9) | 12 (21.8) |  |
| **Mean daily ICS dosage (µg)** | n (% non-missing) | 347 (100.0) | | 47 (100.0) | 161 (100.0) | 55 (100.0) | 0.0133 |
|  | <250 | 103 (29.7) | | 24 (51.1) | 44 (27.3) | 15 (27.3) |  |
|  | 250 to <500 | 91 (26.2) | | 11 (23.4) | 43 (26.7) | 22 (40.0) |  |
|  | ≥500 | 153 (44.1) | | 12 (25.5) | 74 (46.0) | 18 (32.7) |  |
| **Asthma ATS exacerbations** | n (% non-missing) | 347 (100.0) | | 47 (100.0) | 161 (100.0) | 55 (100.0) | 0.0403 |
|  | Mean (SD) | 0.4 (0.7) | | 0.3 (0.5) | 0.4 (0.8) | 0.7 (0.9) |  |
| **Medication cost** | n (% non-missing) | 340 (98.0) | | 45 (95.7) | 158 (98.1) | 55 (100.0) | 0.0062 |
|  | Mean (SD) | 268.5 (228.1) | | 189.6 (222.1) | 285.6 (238.2) | 251.6 (231.6) |  |
| **ICS daily dose** | n (% non-missing) | 347 (100.0) | | 47 (100.0) | 161 (100.0) | 55 (100.0) | 0.0153 |
|  | Mean (SD) | 587.2 (498.6) | | 399.9 (411.7) | 601.5 (498.1) | 555.9 (472.0) |  |
|  | | |  | | |  |  |
| **ICS inhalers** | n (% non-missing) | 347 (100.0) | | 47 (100.0) | 161 (100.0) | 55 (100.0) | 0.0219 |
|  | Mean (SD) | 6.9 (5.1) | | 4.9 (4.0) | 6.7 (4.5) | 6.4 (5.3) |  |
| **ICS prescriptions** | n (% non-missing) | 347 (100.0) | | 47 (100.0) | 161 (100.0) | 55 (100.0) | 0.0100 |
|  | Mean (SD) | 5.4 (3.7) | | 3.8 (3.3) | 5.4 (3.6) | 4.9 (3.6) |  |
| **ICS/LABA prescriptions per patient** | n (% non-missing) | 347 (100.0) | | 47 (100.0) | 161 (100.0) | 55 (100.0) | 0.0056 |
|  | Mean (SD) | 4.1 (4.0) | | 2.6 (3.1) | 4.7 (3.9) | 3.9 (4.0) |  |
| **Asthma related accident and emergency attendance** | n (% non-missing) | 347 (100.0) | | 47 (100.0) | 161 (100.0) | 55 (100.0) | 0.0177 |
|  | Yes | 0 (0.0) | | 0 (0.0) | 0 (0.0) | 1 (1.8) |  |
| **Asthma related unplanned inpatient attendance** | n (% non-missing) | 347 (100.0) | | 47 (100.0) | 161 (100.0) | 55 (100.0) | 0.0002 |
|  | Yes | 0 (0.0) | | 0 (0.0) | 0 (0.0) | 2 (3.6) |  |
| **FEV_1_/FVC ratio** | n (% non-missing) | 45 (13.0) | | 8 (17.0) | 21 (13.0) | 10 (18.2) | 0.0386 |
|  | Mean (SD) | 0.8 (0.1) | | 0.7 (0.1) | 0.7 (0.1) | 0.8 (0.1) |  |
| **Predicted peak flow** | n (% non-missing) | 193 (55.6) | | 33 (70.2) | 79 (49.1) | 40 (72.7) | 0.0038 |
|  | Mean (SD) | 498.0 (73.8) | | 520.8 (67.3) | 533.5 (78.8) | 511.6 (70.0) |  |
| **Percentage predicted peak flow** | n (% non-missing) | 347 (100.0) | | 47 (100.0) | 161 (100.0) | 55 (100.0) | 0.0418 |
|  | Mean (SD) | -66.7 (2103.7) | | 84.5 (17.5) | 91.6 (16.0) | 86.8 (17.9) |  |
| **Rhinitis diagnosis** | n (% non-missing) | 347 (100.0) | | 47 (100.0) | 161 (100.0) | 55 (100.0) | 0.0422 |
|  | Yes | 130 (37.5) | | 13 (27.7) | 74 (46.0) | 27 (49.1) |  |
| **Acute OCS use (yes/no)** | n (% non-missing) | 347 (100.0) | | 47 (100.0) | 161 (100.0) | 55 (100.0) | 0.0486 |
|  | Yes | 93 (26.8) | | 12 (25.5) | 40 (24.8) | 24 (43.6) |  |

All values in the table are n (%) unless otherwise specified. High FeNO defined as ≥35 ppb; non-high FeNO <35 ppb; high blood eosinophil count defined as ≥0.300×10^9^ cells/L; non-high blood eosinophil count <0.300×10^9^ cells/L.

BMI, body mass index; FeNO, fractional exhaled nitric oxide; FEV, forced expiratory volume; FVC, forced vital capacity; GERD, gastroesophageal reflux disease; ICS, inhaled corticosteroid; IHD, ischaemic heart disease; IQR, interquartile range; LABA, long-acting β_2_-agonist; OCS, oral corticosteroids; ppb, parts per billion; SABA, short-acting β_2_-agonist; SD, standard deviation.

Additional file S1: Table 3. Frequency of exacerbations between matched biomarker groups

| **Categorisation 1** | | | |
| --- | --- | --- | --- |
| **Asthma exacerbations** | **Non-high FeNO and non-high blood eosinophils (n=27)** | **High FeNO and high blood eosinophils (n=27)** | **p-value** |
| n (% non-missing) | 27 (100.0) | 27 (100.0) | 0.0427 |
| 0, n (%) | 21 (77.8) | 13 (48.1) |  |
| 1, n (%) | 6 (22.2) | 8 (29.6) |  |
| 2, n (%) | 0 (0.0) | 4 (14.8) |  |
| 3, n (%) | 0 (0.0) | 2 (7.4) |  |
| Mean # exacerbations (SD) | 0.2 (0.4) | 0.8 (1.0) | 0.0109 |
|  |  |  |  |
|  | **Non-high FeNO and non-high blood eosinophils (n=200)** | **High FeNO OR high blood eosinophils (n=200)** |  |
| n (% non-missing) | 173 (100.0) | 173 (100.0) | 0.0481 |
| 0, n (%) | 120 (69.4) | 120 (69.4) |  |
| 1, n (%) | 41 (23.7) | 28 (16.2) |  |
| 2, n (%) | 11 (6.4) | 19 (11.0) |  |
| 3, n (%) | 0 (0.0) | 5 (2.9) |  |
| 4+, n (%) | 1 (0.6) | 1 (0.6) |  |
| Mean # exacerbations (SD) | 0.4 (0.6) | 0.5 (0.8) | 0.3423 |
|  |  |  |  |
| **Categorisation 2** | | | |
|  | **Non-high FeNO and non-high blood eosinophils (n=186)** | **Non-high FeNO and high blood eosinophil (n=186)** |  |
| n (% non-missing) | 186 (100.0) | 186 (100.0) | 0.2149 |
| 0, n (%) | 134 (72.0) | 128 (68.8) |  |
| 1, n (%) | 36 (19.4) | 31 (16.7) |  |
| 2, n (%) | 15 (8.1) | 20 (10.8) |  |
| 3, n (%) | 1 (0.5) | 6 (3.2) |  |
| Mean # exacerbations (SD) | 0.4 (0.7) | 0.5 (0.9) | 0.3134 |
|  |  |  |  |
|  | **Non-high FeNO and non-high blood eosinophils (n=98)** | **High FeNO and non-high blood eosinophils (n=98)** |  |
| n (% non-missing) | 98 (100.0) | 98 (100.0) | 0.3126 |
| 0, n (%) | 72 (73.5) | 63 (64.3) |  |
| 1, n (%) | 20 (20.4) | 24 (24.5) |  |
| 2, n (%) | 6 (6.1) | 9 (9.2) |  |
| 3, n (%) | 0 (0.0) | 2 (2.0) |  |
| 4, n (%) | 0 (0.0) | 0 (0.0) |  |
| Mean # exacerbations (SD) | 0.3 (0.6) | 0.5 (0.7) | 0.1332 |
|  |  |  |  |
|  | **Non-high FeNO and non-high blood eosinophils (n=53)** | **High FeNO and high blood eosinophils (n=53)** |  |
| n (% non-missing) | 53 (100.0) | 53 (100.0) | 0.3080 |
| 0, n (%) | 36 (67.9) | 29 (54.7) |  |
| 1, n (%) | 12 (22.6) | 14 (26.4) |  |
| 2, n (%) | 5 (9.4) | 8 (15.1) |  |
| 3, n (%) | 0 (0.0) | 2 (3.8) |  |
| Mean # exacerbations (SD) | 0.4 (0.7) | 0.7 (0.9) | 0.1166 |

FeNO, fractional exhaled nitric oxide; SD, standard deviation.
